# *Helicobacter pylori* infection induces gastric precancerous lesions and persistent expression of Angpt2, Vegf-A and *Tnf-A* in a mouse model

**DOI:** 10.3389/fonc.2023.1072802

**Published:** 2023-02-15

**Authors:** Wendy Malespín-Bendaña, Warner Alpízar-Alpízar, Lucía Figueroa-Protti, Ledis Reyes, Silvia Molina-Castro, Clas Une, Vanessa Ramírez-Mayorga

**Affiliations:** ^1^ Institute of Health Research (INISA), University of Costa Rica, San José, Costa Rica; ^2^ Centre for Research on Microscopic Structures (CIEMic), University of Costa Rica, San José, Costa Rica; ^3^ Department of Biochemistry, School of Medicine, University of Costa Rica, San José, Costa Rica; ^4^ Faculty of Microbiology, University of Costa Rica, San José, Costa Rica; ^5^ Laboratory for Biological Assays (LEBi), University of Costa Rica, San José, Costa Rica; ^6^ Department Public Nutrition, School of Nutrition, University of Costa Rica, San José, Costa Rica

**Keywords:** *Helicobacter pylori*, angiogenesis, ANGPT1, ANGPT2, VEGFA, mouse model, *TNF-A*

## Abstract

**Introduction:**

*Helicobacter pylori* colonizes the gastric mucosa and induces chronic inflammation.

**Methods:**

Using a mouse model of *H. pylori*-induced gastritis, we evaluated the mRNA and protein expression levels of proinflammatory and proangiogenic factors, as well as the histopathological changes in gastric mucosa in response to infection. Five- to six-week-old female C57BL/6N mice were challenged with *H. pylori* SS1 strain. Animals were euthanized after 5-, 10-, 20-, 30-, 40- and 50-weeks post infection. mRNA and protein expression of Angpt1, Angpt2, VegfA, Tnf-α, bacterial colonization, inflammatory response and gastric lesions were evaluated.

**Results:**

A robust bacterial colonization was observed in 30 to 50 weeks-infected mice, which was accompanied by immune cell infiltration in the gastric mucosa. Compared to non-infected animals, *H. pylori-*colonized animals showed an upregulation in the expression of *Tnf-A*, *Angpt2* and *VegfA* at the mRNA and protein levels. In contrast, *Angpt1* mRNA and protein expression was downregulated in *H. pylori*-colonized mice.

**Conclusion:**

Our data show that *H. pylori* infection induces the expression of Angpt2, *Tnf-A* and Vegf-A in murine gastric epithelium. This may contribute to the pathogenesis of *H. pylori*-associated gastritis, however the significance of this should be further addressed.

## 1 Introduction

Angiogenesis, the process of formation of new blood vessels from the pre-existing, involves proliferation, sprouting and migration of endothelial cells, as well as degradation of the basement membrane. After the re-establishment of cell junctions and coverage of pericytes, the newly formed vessels mature and remain quiescent (1). Importantly, endothelial cells start proliferating to initiate angiogenesis only after stimulation by several growth factors and inflammatory mediators, mainly members of vascular endothelial growth factor (VEGF) family and angiopoietins.

Angiogenesis and tumor promoting inflammation are closely interconnected and are crucial events during cancer progression and metastasis; in fact, they are both considered as hallmarks of cancer (2). Both tumor and stromal cells produce angiogenic factors that ultimately lead to endothelial cell proliferation. These cancer-associated endothelial cells cooperate in the perpetuation of inflammation, which reciprocally promotes angiogenesis by secretion of cytokines, proteases, growth and proangiogenic factors, thus creating a positive feedback loop (1, 2). Aberrant vascular structures can also induce hypoxia, acidosis and DNA damage (3), which contribute to the establishment of a tumor-promoting microenvironment since very early in the carcinogenesis. This could be particularly relevant in cancers in which chronic and persistent inflammation is the main driving force for the malignant transformation, for example in gastric carcinogenesis.

VEGFA is the most potent and ubiquitous member of VEGF family; it is considered as the master inductor of physiological and tumor angiogenesis (4). Secreted by macrophages, lymphocytes, fibroblast and tumor cells, it promotes proliferation, migration and survival of endothelial cells, as well as expression of extracellular matrix proteases (5). Angiopoietin 1 (ANGPT1) and ANGPT2 bind to TIE1-TIE2 (tyrosine kinase with immunoglobulin-like and epidermal growth factor–like domains 1 and 2) receptor complex and α5β1 integrin in endothelial cells, thus activating signaling pathways that lead to blood and lymphatic vessel formation. ANGPT1 is secreted by pericytes and acts in a paracrine manner to keep homeostasis of the mature vasculature. ANGPT2 is mostly produced by endothelial cells, in which it is stored at the cytoplasmatic Weibel-Palade bodies, and released after stimulation, to act in an autocrine way (6). ANGPT2 plays a role as a vessel destabilizing agent that induces permeability and leads to dissociation of cell-cell contacts, allowing the sprouting of new vessels (1, 7). The expression of ANGPT2 in normal tissues is low or absent, but it is upregulated in many cancers in which it is mainly expressed by tumor-associated macrophages (6). In humans, alternative splicing generates a smaller isoform, ANGPT2_443_, which has been reported as upregulated in tumor cell lines and human tumor tissue (8, 9).

TNF-α is a key mediator in a host’s response against gram-negative bacteria, such as *H. pylori*. It is produced by lymphocytes and macrophages and promotes leukocyte recruitment. In the context of angiogenesis, TNF-α primes endothelial cells for angiogenic sprouting by inducing a tip cell phenotype (10).

*Helicobacter pylori* colonization of the gastric mucosa induces chronic and persistent inflammation. This is associated with several clinical outcomes, such as peptic ulcer, MALT-lymphoma and gastric cancer (GC). The latter results from a stepwise cascade of preneoplastic lesions known as Correa’s cascade (11, 12). The persistent inflammation mounted in response to this bacterial infection is characterized by the infiltration in the gastric mucosa of neutrophils, lymphocytes and macrophages. Increased permeability of the endothelium is a pivotal event for the extravasation of immune cells, proteins and fluids (13).

Several reports indicate that *H. pylori* can induce the production of proangiogenic factors *in vitro* and *in vivo* (14–18). Specifically, the expression of HIF-1a, VEFGA and the density of CD31+ blood vessels is higher in *H. pylori*-positive patients, compared to negative persons (19, 20). Those findings suggest that the bacterium is capable of inducing a proangiogenic response after colonizing its host. This could be of pivotal importance during gastric cancer initiation and development. However, the molecules and mechanisms by which *H. pylori* induces early neovascularization in gastric mucosa, and how this influences the precancerous series of events that precede GC is not completely understood.

The present study, we used a mouse model in order to characterize the expression kinetics of *Tnf-A, Angpt1, Angpt2* and *VegfA* in the gastric mucosa in response to *H. pylori* infection and to explore the correlation to inflammation, vascularization and gastric pathology.

## 2 Methods

### 2.1 Animals

**Figure 1:**
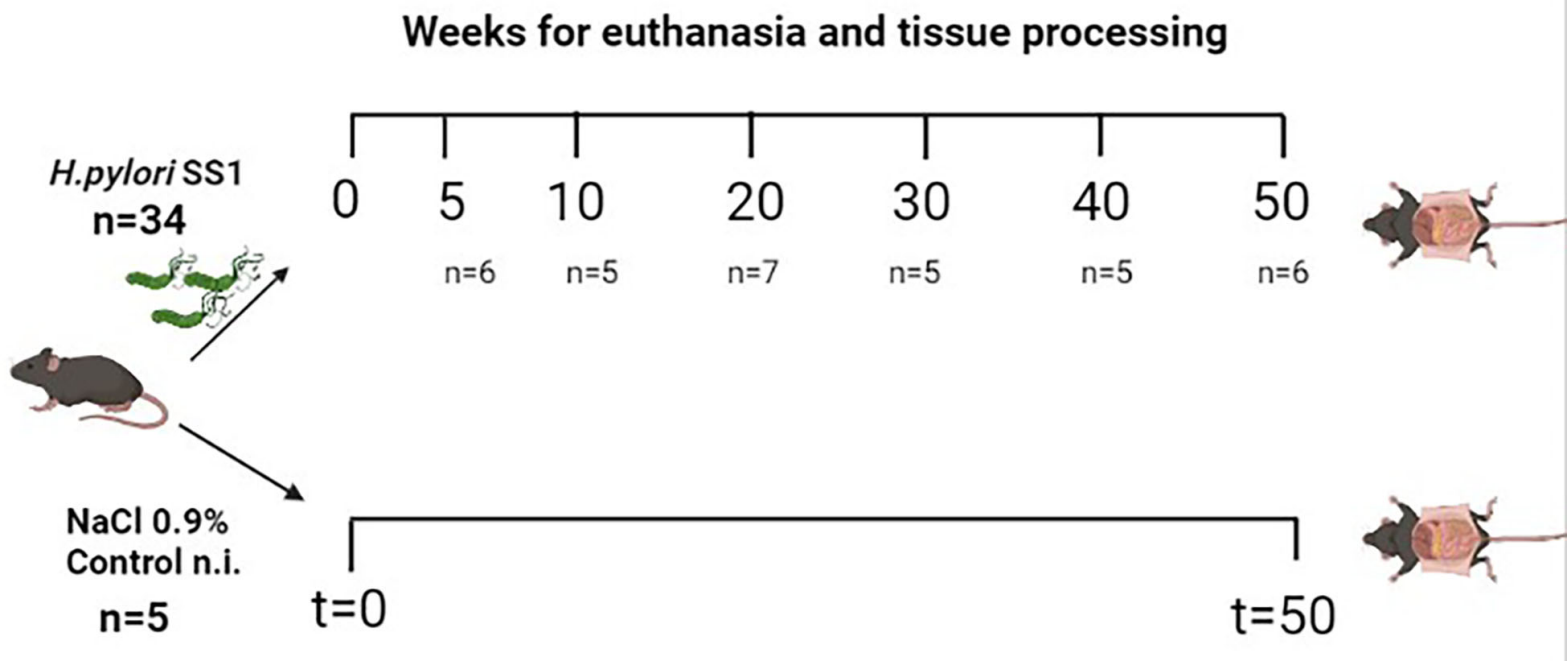
Schematic view of the experimental groups of the mouse model of *Helicobacter pylori* SS1 infection used in the study. A total of 39 female C57BL/6N mice were divided into 7 groups (n per group indicated between parentheses), 6 of these groups were infected with *H. pylori* and one group received NaCl 0.9% only (control group, n=5). Mice were euthanized between 5-50 w.p.i. Control non-infected mice were sacrificed at 50 w.p.i. Created with BioRender.com.

Thirty-nine C57BL/6N female mice certified as *Helicobacter spp*-free were acquired from Harlan Laboratories^®^ and housed at the Laboratorio de Ensayos Biológicos (LEBi), University of Costa Rica, at standard temperature (25.5°C ± 1.20°C) and humidity (50–70%), and maintained on a 12-h light/dark cycle (lights off at 6:00 p.m). Water and food (LabDiet 5010.) were provided *ad libitum*. Mice were distributed into 7 experimental groups, each consisting of 5-7 animals per cage (**Figure 1**) and were moved to an experimental section one week before they were inoculated with *H. pylori*. Experimental procedures and methods were carried out in accordance with the guidelines of the Costa Rican Ministry of Science and Technology and were approved by the Institutional Committee for Animal Care and Use of Animals (CICUA) of the University of Costa Rica (permission number CICUA-031-17).

### 2.2 Bacterial culture and inoculation

The inoculation was performed with SS1 strain (mouse-adapted cagA+, vacA+ strain with no functionality of the cag pathogenicity island; kindly donated by Dr. James Fox, MIT, USA). Bacteria were grown on Skirrow plates and incubated at 37°C for 5-7 days under microaerophilic conditions. The SS1 strain was harvested and mice were inoculated by oral gavage with a single dose of approximately 10^9^ bacteria (CFU) in 0.2 mL of 0.9% NaCl. Control mice received 0.2 mL of the saline solution only. The bacteria used for inoculations were isolated from previously colonized mice from our bioterium.

### 2.3 Resection and processing of gastric tissue for histology

Infected groups were euthanized by cervical dislocation after 5-, 10-, 20-, 30-, 40- and 50- weeks post-infection (w.p.i.). The control group was euthanized at 50 w.p.i. Entire stomachs were opened along the greater curvature, washed with PBS, and cut longitudinally into four stripes, extending from the squamous forestomach through the duodenum. The stripes were used as follows: one for confirmation of bacterial colonization (culture), one was cut in halves for RNA and protein isolation, and two for immunohistochemistry assays.

### 2.4 Culture for confirmation of *H. pylori* colonization

The stripe was macerated with saline solution, vortexed 5 s, then 40 μl of the suspension was seeded in both Skirrow and agar-blood BHI plates and incubated at 37°C for 5-7 days under microaerophilic conditions.

### 2.5 Quantitative real time PCR

The stomach tissue was suspended in RNAlater (Invitrogen) until utilization. Total RNA was isolated with Trizol (Invitrogen) in accordance with the manufacturer’s instructions. RNA from each sample was transcribed into cDNA using High-capacity cDNA reverse transcription kit (ThermoFisher Scientific). A total of 0,5 μl cDNA was used for qRT-PCR amplification using TaqMan Gene Expression Assays (Thermo Fisher Scientific) for *Angpt1* (Mm00456503_m1)*, Angpt2* (Mm00545822_m1)*, Tnf-A* (Mm00443258_m1) and *VegfA* (Mm00437306_m1) with *Gapdh* (Mm99999915_g1) as endogenous control, using the StepOne Plus thermal cycler (Applied Biosystems, Foster City, CA, USA) under the following conditions: 95°C for 10 sec, 95°C for 5 sec, and 60°C for 30 sec, run for 40 cycles. Fold-difference for infected *vs*. non infected mice was estimated by the 2 ^-ΔΔCt^ method.

### 2.6 Western blot analysis

Stomach tissues were macerated and lysed using lysis buffer solution (PBS 1X, 1% Triton X-100, 1% NP-40, proteases and phosphatases cocktail inhibitors, pH 7.4). Following centrifugation at 14 000 g for 30 min, the proteins were quantified using the Protein Assay Dye Reagent Concentrate (BioRad) and 50 µg of protein were separated by 10% SDS−PAGE, prior to being transferred onto a nitrocellulose membrane (BioRad). Following blocking with 5% fat−free milk in PBS buffer in 0.05% Tween 20 for 1 h at room temperature, the membranes were then separately incubated overnight at 4˚C with the following monoclonal antibodies: Rabbit ANGPT1 (1:500; Abcam ab102015), Rabbit ANGPT2 (1:500, Abcam ab8452) and Rabbit GAPDH (1:1000, Abcam, ab9485). The secondary antibodies were applied (dilution 1:10 000, horseradish peroxidase-conjugated anti-rabbit, Sigma-Aldrich A9169) at room temperature for 1 h. Labeled bands were detected by enhanced chemiluminescence (Clarity ECL, BioRad, USA) and analyzed by Chemidoc Imaging System (Bio-Rad). Protein levels were quantified using the Image Lab software, version 4.1 (Bio-Rad, USA).

### 2.7 Immunohistochemical detection of Angpt1, Angpt2, VegfA, CD31 and *H. pylori*

The stinging protocols were a modification of Alpízar-Alpízar et al. (21). Two stomach stripes were fixed 24 hours in 4% paraformaldehyde and paraffin embedded. Then, 4 μm tissue sections were deparaffinized in xylene and hydrated in a gradual series of ethanol-water dilutions. For *H. pylori*, sections were pretreated with Proteinase K (10 μg/mL) for 15 min, 37°C. For CD31, Angpt1, Angpt2 and VegfA, sections were pretreated at 98°C for 15 min in 10 mM sodium citrate pH 6.0. In all cases, endogenous peroxidase activity was blocked by incubation in 3% H_2_O_2_ solution for 15 min. The primary antibodies were diluted in Antibody Diluent (Dako, code S3022) and incubated overnight at 4°C in Shandon racks (Thermo Shandon, Pittsburg, PA, USA) at the following dilutions: Rabbit-anti *H. pylori* 1:150 (Dako, code B0471), Rabbit anti-Angpt1 1:500, Rabbit anti-Angpt2 1:1000, Rabbit anti-CD31 1:250, Rabbit anti-VegfA 1:200. All primary antibodies were detected with EnVision reagent anti-rabbit IgG horseradish peroxidase-conjugated polymers (Dako Code: K3468). Each incubation step was followed by washes in TBS containing 0.5% (v/v) Triton X-100. Finally, the reactions were visualized by incubating the sections with Liquid DAB+ substrate chromogen system (Dako, Code S3022) and counterstained with Mayer’s hematoxylin.

### 2.8 *H. pylori* colonization, histopathology and immunohistochemistry evaluations

*H. pylori* colonization was evaluated according to the number of gastric glands containing bacteria and their density, as follows: 0: No observed bacteria; 1: Occasional pits and/or glands with individual bacteria; 2: frequent pits and/or glands with individual bacteria; 3, infrequent pits and/or gland with dense bacterial colonies; and 4, frequent pits and/or glands with dense bacterial colonies (22). Sections were stained with hematoxylin and eosin (H&E) for histopathological evaluations of the mouse gastric mucosa describing inflammation, infiltration, metaplasia, and anatomical localization of the lesions, using published guidelines (23): 0, no infiltration, 1, patchy or multifocal small islands of inflammatory cells in the mucosa and/or submucosa; 2, coalescing aggregates of inflammatory cells in submucosa or mucosa; 3, organizing nodules of lymphocytes and other inflammatory cells in submucosa and mucosa; 4, follicles and/or sheets of inflammatory cells extending into or through muscularis propria  adventitia. Photomicrographs were captured using the bright field microscope Motic BA400 and Motic Images Plus 3.0 software (China).

### 2.9 Statistical analysis

Data for gene expression is represented as the mean ± standard deviation for every experimental group. Data for inflammation and *H. pylori* scores are represented with median values. The association between variables on an ordinal scale is presented by the Spearman rank correlation. Experimental data were compared among the groups using the Kruskal-Wallis test. p-values less than 0.05 were considered significant. ***p < 0.001. Data presented as mean ± SD of each experimental group. All data were analyzed using the GraphPad Prism 9 software.

## 3 Results

### 3.1 Histopathological changes induced by *H. pylori* infection

**Figure 2:**
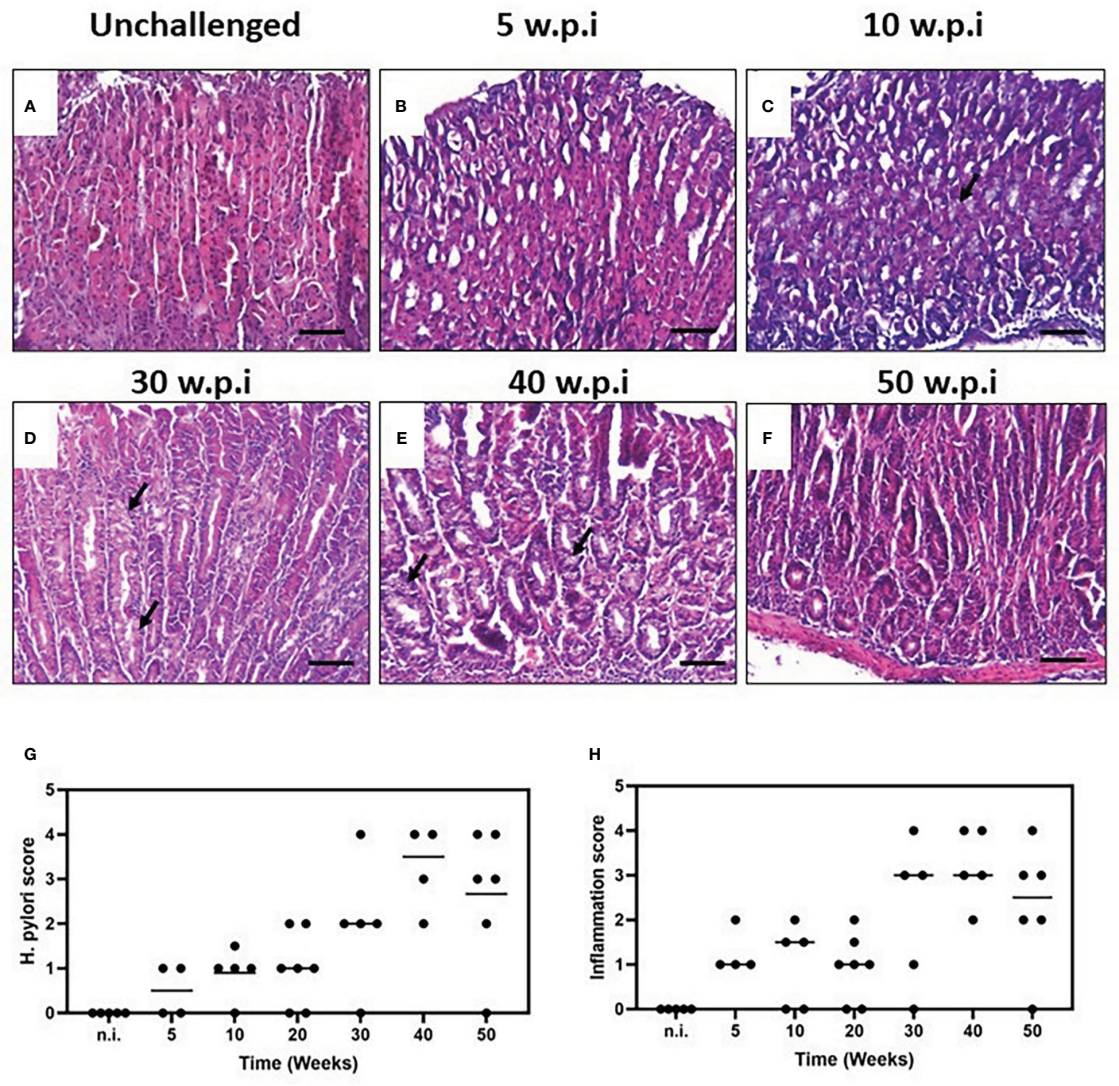
Histopathology of the murine gastric corpus mucosa in unchallenged and *H. pylori*-challenged mice. Tissue sections from resected gastric mucosa of unchallenged and H. pylori-inoculated mice were stained by H&E. **(A)** Unchallenged mice show a normal mucosal architecture. **(B)** Five weeks after *H. pylori* inoculation, minor multifocal infiltration of immune cells is observed. **(C)** Minor infiltration and some metaplastic patches are observed ten weeks after challenge. **(D)** The pathology and infiltration are intensified after thirty weeks of infection, with hyperplasia and multiple foci of mucous metaplasia. **(E)** Animals infected for forty and fifty **(F)** weeks also showed an extensive infiltration of immune cells with areas of hyperplasia and metaplasia (black arrows). *H. pylori* colonization status and histopathological assessment of inflammation. **(G)** *H. pylori* score at different time-points. **(H)**. Inflammation score of the murine gastric mucosa with *H. pylori* infection at different time-points (scored according to the scheme proposed by Wang et al. (22) and Rogers (23), one experiment; n = 5-7 mice per point; black line represents median values). Formalin-fixed, paraffin-embedded gastric tissue were stained with hematoxylin and eosin and examined. Scale bars: 50 μm (20x).

In the present study, we used a mouse model of *H. pylori*-induced gastritis to explore the relationship between the bacterium and the induction of proangiogenic and inflammatory mediators *in vivo*. We evaluated the histopathological changes and lesions induced by the *H. pylori* infection in the groups of challenged and unchallenged mice. Non infected mice showed a normal mucosal architecture (**Figure 2A**). Five weeks after colonization, *H. pylori*-infected mice showed scattered immune cells infiltrating mucosa and submucosa, at antrum, corpus and the squamo-columnar transition. Two of the animals showed patches of mucous metaplasia in corpus (**Figure 2B**). Ten weeks after challenge, mice showed minor immune cell infiltration at the mucosa, especially at the squamo-columnar and corpus-antrum transitions, with some mucous metaplastic patches (**Figure 2C**). Twenty-week-colonized mice showed minor immune cell foci in antral mucosa, at the base of the corpus glands and squamo-columnar transition (not shown). Thirty weeks after inoculation, mice presented a stronger and widespread infiltration of immune cells in mucosa and submucosa of corpus and corpus-antrum transition, with hyperplasia and multiple foci of mucous metaplasia (**Figure 2D**). Forty- and fifty-week challenged animals also showed an abundant and extensive immune cell infiltration in mucosa and submucosa of the corpus, with large areas of hyperplasia and mucous metaplasia (**Figures 2E, F**).

### 3.2 Description of the *H. pylori* colonization score on gastric mucosa

We assessed the *H. pylori* colonization status by culture and immunohistochemistry for each animal and found *H. pylori*-positive mice in all time points. In accordance with the bacterial score, the most robust infiltration of immune cells and more severe lesions were found at 30, 40 and 50 w.p.i (**Figures 2G, H**), p=0.0187).

### 3.3 *H. pylori* infection induces concomitant expression of *Tnf-A*, *Angpt2* and *VegfA*

**Figure 3:**
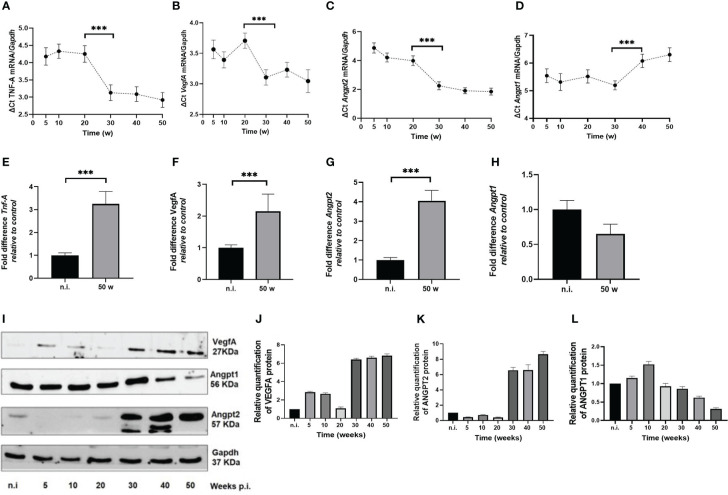
*H. pylori*-SS1 upregulates the expression of pro-angiogenic factors in murine gastric mucosa. mRNA expression was analyzed by real-time PCR, and represented as Delta (Δ) Ct values. The higher ΔCt values represents the lower expression of genes. **(A)** *Tnf-A*, **(B)** *VegfA* and **(C)** *Angpt2* mRNAs show a concomitant upregulation in time, becoming more evident from 30 w.p.i, whereas **(D)** *Angpt1* mRNA is downregulated at 40 and 50 w.p.i. When comparing not infected animals with the 50 w.p.i. group, a significant upregulation was observed for **(E)** *Tnf-A*, **(F)** *VegfA* and **(G)** *Angpt2* mRNAs expression, but a significant deregulation for **(H)** *Angpt1*mRNA. **(I-L)** Western blot and concordant relative quantification of VegfA, Angpt1 and Angpt2 proteins in mouse gastric mucosa of *H. pylori*-SS1 infected and uninfected mice are shown. VegfA and Angpt2 are upregulated and Angpt1 is downregulated in time. Data presented as mean ± SD of each experimental group. ***p < 0.001.

We assessed the expression at the mRNA and protein level of several proangiogenic factors in the gastric mucosa in our mouse model. In the *H. pylori*-infected animals *Tnf-A*, *VegfA* and *Angpt2* mRNAs normalized to *Gapdh* showed an increased expression in time, showing the lowest ΔCt values at 30 to 50 w.p.i (**Figures 3A–C**). When comparing unchallenged animals with the 50 w.p.i. group, a significant upregulation was observed for *Tnf-A, VegfA* and *Angpt2* mRNAs expression (**Figures 3E–G**)

### 3.4 *Angpt1* mRNA exhibited a peculiar expression

In *H. pylori*-infected mice from 5 to 20 w.p.i., there was a slight increment that reached its top level of expression at 30 w.p.i., but at 40 and 50 w.p.i. the mRNA levels were drastically downregulated (**Figure 3D**). At 50 w.p.i *Angpt1* mRNA levels were significantly different from the not infected animals (**Figure 3H**). When analyzing the expression of *Tnf-A, VegfA, Angpt2* and *Angpt1* altogether, it is worth nothing that *Tnf-A, Vegf-A* and *Angpt2* mRNAs showed a significant upregulation starting at 30 w.p.i, that coincides with the downregulation of *Angpt1* at the 40 and 50 w.p.i. This is important since all these molecules act in concert to trigger the angiogenic switch.

**Figure 4:**
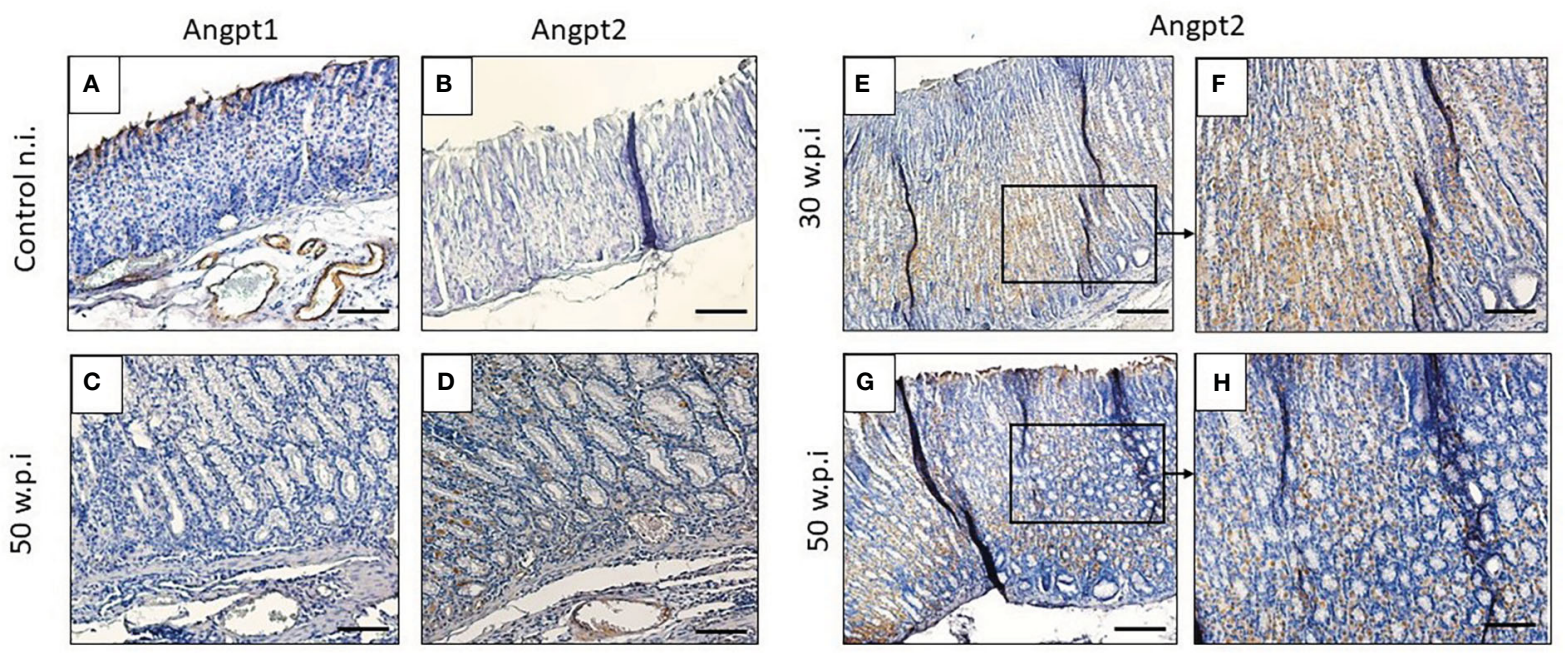
*H. pylori* upregulates Angpt2 expression in murine gastric mucosa. **(A, B)** IHC staining in blood vessels is positive (brown color) for Angpt1 and negative for Angpt2 in unchallenged mice. **(C, D)** IHC staining of Angpt1 compared to Angpt2 in lamina propria and blood vessels of infected mice 50 w.p.i. IHC staining with Angpt2 in infected mice 30 w.p.i. compared to 50 w.p.i. mice. Scale bars: **(A, B, E, G)** ≈ 50 μm; **(C, D, F, H)** ≈ 100 μm.

The Western blot analysis showed an upregulation of VegfA and Angpt2 proteins at 30 to 50 w.p.i (**Figures 3F, G, I**). In contrast, Angpt1 protein was downregulated (**Figures 4F, H**). Of note, two bands for Angpt2 protein with weights between 50-57 KDa, were detected in 30 and 40 w.p.i mice. This was not observed at 50 w.p.i. (**Figure 3F**).

We also performed immunohistochemistry to assess the expression pattern and localization of Angpt1 and Angpt2 in murine tissue. *H. pylori* infection induced Angpt2 expression in gastric epithelial cells. Unchallenged mice showed a strong staining for Angpt1 in blood vessels, but Angpt2 was not detected (**Figures 4A, B**). Angpt2 immunoreactivity was observed in gastric mucosa of infected mice, with an intense expression in metaplastic areas at the corpus mucosa in 30- and 50-w.p.i. challenged mice (**Figures 4D–G**), but no signal for Angpt1 (**Figure 4C**).

## 4 Discussion

Pathological angiogenesis is a process that takes place during chronic inflammation and cancer. It involves activation of endothelial and immune cells that secrete pro-angiogenic factors such as VEGF-A, ANGPT2, TNF-α and MMPs, thus triggering proliferation of quiescent endothelium to make new blood vessels. This has been intensively studied in several cancer types, including GC. Although some evidence from *in vitro* models and studies in human gastric mucosa suggest that *H. pylori* infection may be involved in the induction of pro-angiogenic factors as VEGFA and HIF-1 (17–19, 24–26), to the best of our knowledge, no studies have addressed the role of *H. pylori* infection in the induction of proangiogenic proteins as Angpt1 and Angpt2 in the gastric mucosa. Therefore, we used a mouse model of *H. pylori*-induced gastritis to explore the kinetics of the expression of Angpt1, Angpt2, Tnf-α and Vegf-A *in vivo*.

We found a significant upregulation of *Angpt2* mRNA and protein, whereas *Angpt1* mRNA was downregulated. The ANGPT2/ANGPT1 balance determines the fate of the endothelium. ANGPT2 has opposing role to ANGPT1 since it promotes blood vessel wall destabilization. This is achieved by competitively inhibiting the binding of ANGPT1 to Tie-2 and reducing Tie-2 activation and phosphorylation (1). ANGPT2 is produced by endothelial cells and macrophages, and plays a key role in the promotion of vessel sprouting, pericyte detachment and basement membrane remodeling. ANGPT1 is produced by pericytes and is associated with endothelium quiescence. ANGPT2/ANGPT1 imbalance parallels capillary destabilization; as the inflammatory response mounted against *H. pylori* infection persists, activated endothelial and infiltrating immune cells produce increasing ANGPT2 levels while ANGPT1 is downregulated, ultimately leading to vessel destabilization (1, 6).

In mouse and human, the *Angpt2/ANGPT2* gene encodes a 496 aa protein of 57 KDa. Hypoxia is an inducer of alternative splicing in cancer and endothelial cells that frequently display a temporal and tissue-specific expression (27). In humans, alternative splicing generates the ANGPT2_443_ isoform lacking exon 2, which produces a smaller (443 aa) 51 KDa protein that is found in activated endothelial cells and macrophages. Reports indicate that functions as a more potent competitive inhibitor of ANGPT1 (8) and is upregulated in cancer cell lines, breast tumor tissues, and canine adrenocortical tumor tissue (27, 28). In the performed Western blots we observed the presence of two bands with weights between 50-57 KDa for the infected mice of groups of 30 and 40 w.p.i. (**Figure 3F**), using an antibody (ab8452, Abcam) predicted to recognize both isoforms of Angpt2. We hypothesize that the observed bands could be Angpt2_443_. Nevertheless, as there are no previous reports for the expression of the isoform Angpt2_443_ in mice in normal or pathological conditions, further transcriptomic and protein assays are necessary to check whether the observation of the present study corresponds to that isoform.

We observed a concomitant upregulation of *Tnf-A, Vegf-A, Angpt2* mRNAs at 30, 40 and 50 w.p.i. *In vitro* studies have revealed that TNF-α upregulates the expression of *VEGF-A*, *ANGPT2*, and *Tie2*, and deregulates *ANGPT1* genes at mRNA level (29, 30). After translation, the ANGPT2 protein is stored in Weibel-Palade bodies at endothelial cells. The main factor causing its secretion is TNF-α, which ultimately results in autocrine inhibition of the Tie-2 receptor (31). TNF-α is mainly produced by activated macrophages and T lymphocytes, and is a potent activator of endothelium by inducing vasodilatation, increase of vascular permeability and the recruitment of immune cells (32). TNF-α expression is substantially elevated in response to *H. pylori* infection thus inhibiting gastric acid secretion, which in turn facilitates *H. pylori* survival (32). *TNF-A* transcription is induced by nuclear factor κappa B (NF-κB), which is activated in response *H. pylori* infection in gastric epithelial cells *in vitro* and *in vivo* (15, 33, 34), and upregulates VEGFR in endothelial cells (16).

Besides its role in cancer progression, angiogenesis plays a major role in the multi-step carcinogenesis process due to the fact that the gastric mucosa undergoes important histological changes, which may require gaining access to the vasculature in order to receive an adequate supply of nutrients and oxygen (35). Tuccillo et al. (36) found elevated levels of VEGFA in *H. pylori*-associated human gastritis. VEGFA and iNOS levels are also high in patients with chronic atrophic gastritis as well as in metaplastic and dysplastic areas (37). These observations, and ours, support the evidence pointing to the role of *H. pylori* as a promotor of angiogenesis.

**Figure 5:**
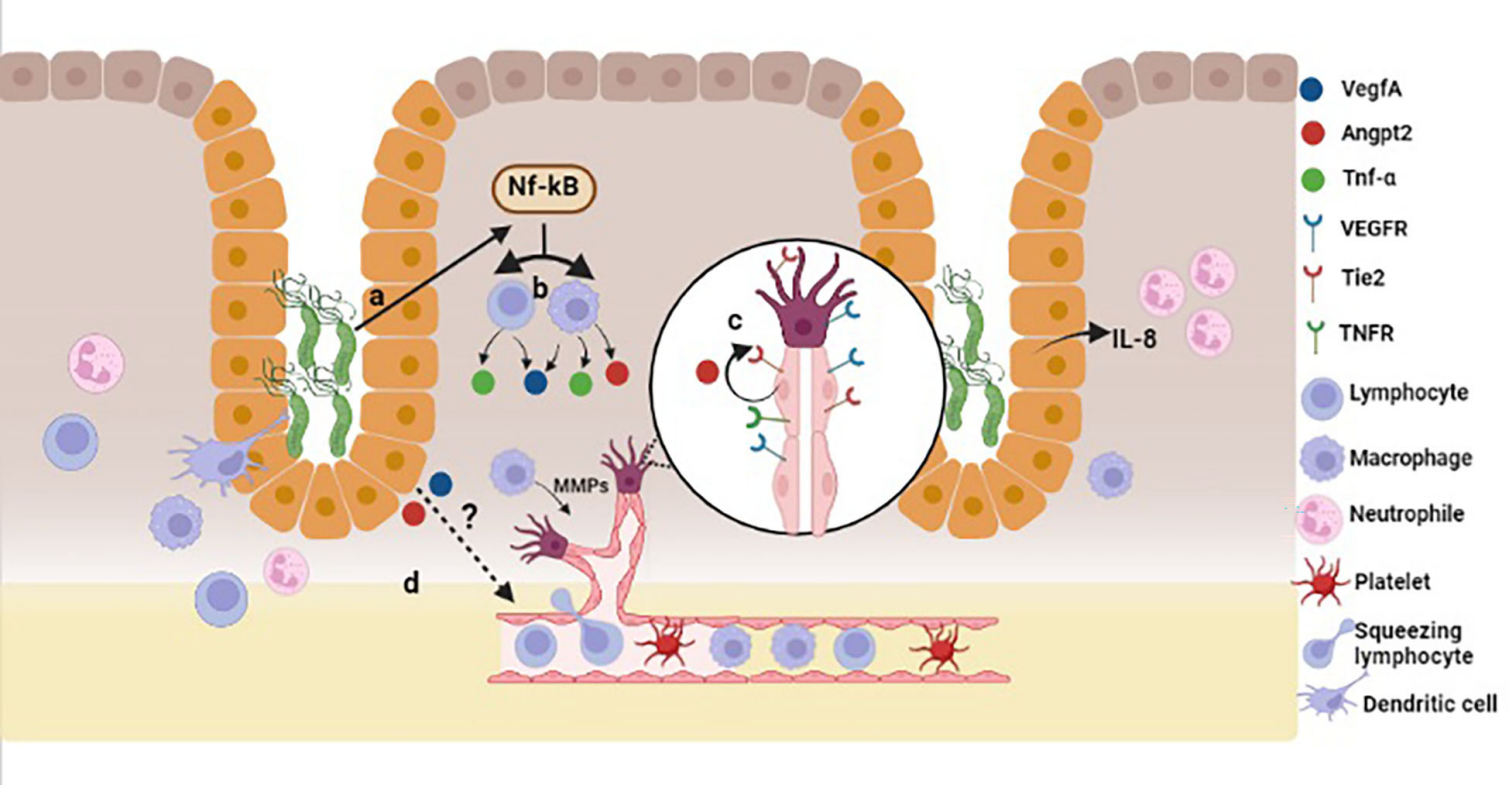
Model for *H. pylori*-induced angiogenesis in gastric mucosa. In a CagA-independent pathway, *H. pylori* infection may trigger the activation of NF-κB **(A)**, which in turn induces TNF-α production **(B)** in lymphocytes and macrophages. TNF-α activates endothelial cells, which promotes Angpt2 release **(C)** from Weibel-Palade bodies. *H. pylori* induces the expression of *Angpt2* and *VegfA* from epithelial cells in a CagA-independent pathway, and also the activation and proliferation of endothelial cells **(D)**. Created with BioRender.com.

The normal gastric epithelium does not express angiogenic factors; those are secreted by endothelial and some immune cells under stimulus. Several *in vitro* studies, including our own observations (manuscript submitted), show that in co-cultures *H. pylori* induces the expression of *ANGPT2*, *VEGFA* and other angiogenesis-related factors in gastric adenocarcinoma cell lines (17, 26, 38). Atrophic, hyperplastic, metaplastic or dysplastic cells in *H*. *pylori*-infected gastric mucosa have accumulated genetic and epigenetic alterations that may change their gene expression programs thus leading to alteration in their phenotypes (39). In this context, it could be possible that the gastric epithelial cells themselves secrete some angiogenic factors, in response to signals from the inflammatory microenvironment, bacterial virulence factors (e.g. CagA, VacA, LPS, urease), or both (**Figure 5**). In the present study we challenged mice with *H. pylori* SS1, which has a nonfunctional CagA protein. Therefore, the mechanisms by which the bacterium induces angiogenic responses may be CagA-independent. Other virulence factor, such as urease, may be implicated since it has been demonstrated that this enzyme induces ANGPT2 and other angiogenic factors in human gastric cell lines (17).

In conclusion, in this study we have found that *H. pylori* infection concurrently modulates the expression of several angiogenic mediators in the murine stomach. Specifically, this bacterial infection induces the expression of Angpt2, *Tnf-A* and VegfA at the mRNA and protein levels. Concomitantly, it downregulates Angpt1 expression in mouse gastric mucosa in a time-dependent manner. Nevertheless, our study has two major limitations; first, we had only one group of non-infected mice for the experiment that was euthanized at 50 weeks, but no control groups for early time points of infected animals. Hence, we show only the evolution of the expression in infected animals, and the comparison with the control group is shown only for the 50 w.p.i. mice. Second; the presented evidence describes the role of *H. pylori* in the modulation of the studied angiogenic factors, therefore, subsequent mechanistic and functional studies are necessary to establish the impact to *H. pylori* infection to angiogenesis *in vivo*.

## Data availability statement

Datasets are available on request: The raw data supporting the conclusions of this article will be made available by the authors, without undue reservation.

## Ethics statement

The animal study was reviewed and approved by Institutional Committee for Animal Care and Use of Animals (CICUA) of the University of Costa Rica (permission number CICUA-031-17).

## Author contributions

VR-M, WA-A, WM-B, CU, SM-C and LR designed the study. WM-B, WA-A and LF-P performed the experiments. WM-B, WA-A, VR and SM-C analyzed the data. WM-B, VR and WA-A interpreted the data. WM-B, WA-A and VR wrote the manuscript. All authors contributed to the article and approved the submitted version.
